# The Influence of High-Intensity Ultrasonication on Properties of Cellulose Produced from the Hop Stems, the Byproduct of the Hop Cones Production

**DOI:** 10.3390/molecules27092624

**Published:** 2022-04-19

**Authors:** Monika Szymańska-Chargot, Jolanta Cieśla, Patrycja Pękala, Piotr M. Pieczywek, Wiesław Oleszek, Marcin Żyła, Zbigniew Szkopek, Artur Zdunek

**Affiliations:** 1 Institute of Agrophysics, Polish Academy of Sciences, Doświadczalna 4, 20-290 Lublin, Poland; j.ciesla@ipan.lublin.pl (J.C.); p.pekala@ipan.lublin.pl (P.P.); p.pieczywek@ipan.lublin.pl (P.M.P.); a.zdunek@ipan.lublin.pl (A.Z.); 2 Department of Biochemistry, Institute of Soil Science and Plant Cultivation, State Research Institute, 24-100 Puławy, Poland; wo@iung.pulawy.pl; 3 Energy Composites Ltd., Marklowicka 30A, 44-300 Wodzisław Śląski, Poland; marcin.zyla@e-composites.eu (M.Ż.); zbigniew.szkopek@alumast.eu (Z.S.); 4 Polski Eko Chmiel Inc., Tomasza Zana 11a, 20-601 Lublin, Poland

**Keywords:** hop stems, hop byproducts, cellulose, nanocellulose

## Abstract

The goal of this work is to evaluate the hop stems, a byproduct of hop cones production, as a potential source of cellulose. Hop stems contain up to 29% of cellulose. The cellulose isolation was conducted through the thermochemical treatment. After high-speed blending, the cellulose was characterized by 67% of crystallinity degree obtained from X-ray diffraction and median diameter of 6.7 nm obtained from atomic force microscopy imaging. The high-intensity ultrasonication (HIUS) was applied to reach further disintegration of cellulose fibers. The longer HIUS treatment resulted in decrease in crystallinity degree even up to 60% and decrease in the fiber diameter up to 4 nm. The Fourier transform infrared spectroscopy (FTIR) spectra showed that HIUS treatment led to changes in intermolecular hydrogen bonds. The stability of cellulose dispersions versus length of HIUS treatment was monitored over 14 days with back dynamic light scattering and laser Doppler electrophoresis methods. Obtained results are evidence that the hop stems are a potential source of cellulose and that it is possible to obtain stable dispersions after HIUS treatment. This was the first time that the properties of hop cellulose have been described so extensively and in detail after the use of HIUS treatment.

## 1. Introduction

Hop (*Humulus lupulus* L.) is one of the *Cannabaceae* plant family [1]. For centuries, hop has been mainly cultivated for its cones, which are used in the brewing industry. Annual hop cones production is about 50,000 tons per year in the EU alone [2]. The remaining leaves and stems are considered as byproducts with limited application, and often they are just burned, contributing to the emission of pollutants. In the best case, they are composted and used as fertilizer [1]. It is assumed that byproducts of hop cultivation constitute two-thirds of total production, which gives about 100,000 tons per year in the EU alone.

Over the last two decades the agriculture byproducts have been investigated for the possibility to become a cheap, renewable and abundant source of cellulose and also nanocellulose [1,3]. Most of those byproducts consist of 30–70% of cellulose, which can find its application as a high-value additive for biopolymers [3]. Hemp, which is also a member of *Cannabaceae* family, is a well-known source of textile fiber that contains of 68% of cellulose, while information about hop stems as a source of cellulose is scarce [1,3].

Cellulose is a polymer of ß-(1,4)- D glucose which is self-assembled into microfibrils and is one of the major constituents of plant cell walls [4]. Cellulose microfibrils are heterogenous and consist of highly ordered (crystalline) parts altered with amorphous ones. Cellulose macroscopic structure such as crystallinity degree, microfibrils width or length depends on plant origin [5,6,7]. The other components of plant cell walls are pectins, hemicelluloses, lignin and, in low amounts, phenolic compounds and proteins [8]. Therefore, cellulose isolation is mainly based on the process of removing these components [9]. Usually, this is attained by a thermochemical method involving acid and/or alkali treatment which removes pectic and hemicellulosic polysaccharides. This is followed by a bleaching process with active chlorine and oxygen to remove lignins [6]. 

The structure of cellulose as the main component of cell wall scaffolding has tremendous effect on mechanical properties of plant tissue and thus on mechanical properties of the whole plant. This is especially important in the case of climbing plants such as hop plants. As it was mentioned above, hop stems so far treated as waste can be valorized as a source of cellulose. Then after preprocessing of cellulose, nanocellulose can be prepared which can be used as a reinforcing agent of many composites used as packaging materials or construction elements. One of the methods of preprocessing of cellulose which leads to the nanocellulose production is high-intensity ultrasonication. Generally, nanocellulose is all cellulosic particles with at least one dimension in nanometers. Those nanomaterials are categorized into two groups: cellulose nanocrystals (CNC) and cellulose nanofibrils (CNF). Cellulose nanocrystals are prepared via chemical treatment, i.e., strong acid hydrolysis of cellulose amorphous parts. This process leaves short and thin parts (less than 1µm in length and several nm width) of highly crystalline (up to 90% of crystalline parts) cellulose [3]. Cellulose nanofibrils are prepared by mechanical processing such as microfluidization, grinding, cryo-crushing, high-speed blending, or high-intensity ultrasonication, giving long (micrometer scale), thin (from several up to tens of nm) and flexible nanofibrils with a lower crystallinity degree than cellulose nanocrystals [3,6]. 

Taking into account the substantial amount of hop byproducts to be managed annually, the aim of this paper is to evaluate hop stems as a source of highly valuable cellulose. The cellulose was isolated from hop stems, and its physicochemical properties were characterized. Then the influence of high-intensity ultrasonication was applied to isolated cellulose. The influence of process conditions (time of ultrasonication treatment) of isolation on cellulose properties was also evaluated.

## 2. Results

### 2.1. Characterization of Hop Stems

**Figure 1:**
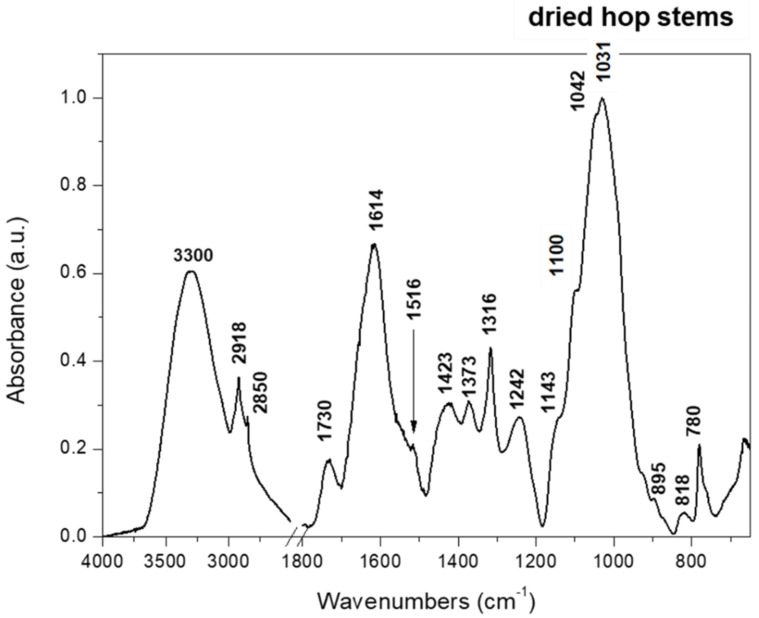
FTIR spectrum of dried raw hop stems in range 4000—650 cm^−1^ (region 2750–1800 was cut out due to lack of spectral information).

To better understand the composition of raw hop stems the FTIR spectrum was collected (Figure 1). Spectrum of dried raw hop stems shows overlapping bands characteristic for typical plant cell wall components. The region of 3750–2500 cm^−1^ is dominated by O-H, C-H and C-H_2_ vibration characteristic for both polysaccharides and lignin. Lignin is also characterized by bands at 1730 cm−1 (phenolic esters, but as well as esterified uronic acids) or 1516 cm^−1^ (phenolic ring, C=C) [10]. In region 1750–1400, bands at 1639 cm^−1^ (phenolic ring, C=O), 1612 cm^−1^ (phenolic ring, C=C) and 1430 cm^−1^ (phenol), characteristic for lignin, are also overlapped by bands typical for nonesterified uronic acids present in pectic polysaccharides (-COO-, 1614 and 1423 cm^−1^) [10,11]. Hemicelluloses are represented mainly by bands at 1373 and 1316 cm^−1^ (xyloglucan, CH_2_ bending), while cellulose bands at 895 and 1031 cm^−1^ are connected with stretching vibration of C-C and C-O, and bending of ß-glycosidic linkage, respectively [12]. The band with maximum at 1031 cm^−1^ is a superposition of bands characteristic also for pectic and hemicellulosic polysaccharides [13].

Additionally, the hemicellulose, cellulose and lignin content in hop stems residue was characterized by the Van Soest method and expressed as g/100 g of dry matter [9,14]. The dry matter of hop stems material contained 48.93 g/100 g d.m. of neutral detergent fiber (NDF), which was composed of 10.94 g/100 g d.m. of hemicellulose, 8.93 g/100 g d.m. of lignin and 29.06 g/100 g d.m. of cellulose (Table 1). The rest (51.07 g/100 g d.m.) was the neutral detergents soluble (NDS) which usually contain phenolic compounds, proteins, sugars and small oligosaccharides. Overall dry matter content of hop stems was 27.23 ± 0.06%. So far, this is the first time that cellulose content in raw and unprocessed hop stems was evaluated. Previously, Reddy and Yang (2009) showed that hop fibers extracted with sodium hydroxide and then with a mixture of chromic and nitric acids contained 84% of cellulose, which was comparable with cellulose content in cotton [1]. It should be stressed that high cellulose content was the result of hop stems digestion leading to obtaining single fibers containing high content of cellulose bundles connected together with lignin. 

The content of cellulose in hop stems obtained in this study is generally lower compared with other residues such as corn stalks (41–48%), rice straw (52.3%), barley straw (48.6%), flax shives (39.9%), hemp (55–77%) or kenaf (45–47%) [6,15,16,17]. However, compared with those residues, hop stems contained relatively small amounts of hemicelluloses and lignin. 

### 2.2. Characterization of Cellulose and Nanocellulose Obtained from Hop Stems

Cellulose from hop stems was isolated by thermochemical method (Supplementary Materials Figure S1a). The influence of duration of high-intensity ultrasonication treatment on cellulose structure was also investigated resulting in acquisition of several dispersions of nanocellulose (0.1%HIUS_180, for example, Supplementary Materials Figure S1b). In the case of initially dispersed sample by Ultra Turrax (0.2%UT, Supplementary Materials Figure S1b), sedimentation occurs, while after HIUS treatment the cellulose dispersions are stable for a long time (0.1%HIUS_180, for example, Supplementary Materials Figure S1b). The structure of obtained samples was characterized in terms of morphology, crystallinity degree, thermal properties and molecular structure.

**Figure 2:**
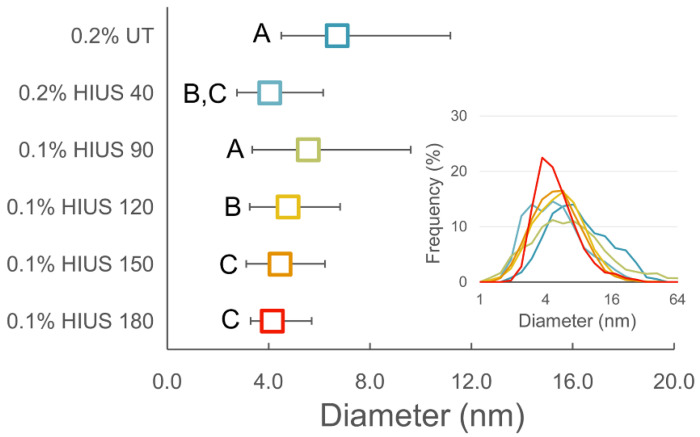
Changes in diameter of cellulose fibers with respect to applied ultrasound treatment. Points on the main plot indicate median values of fiber diameters, while whiskers indicate lower (Q1) and upper quartile (Q3), respectively. Subplot shows distribution of fiber diameters in logarithmic scale; different letters mean the significantly different results at *p* < 0.05.

Influence of HIUS treatment on cellulose morphology, i.e., diameter, is presented in Figure 2 and Supplementary Materials Figure S2. With the exception of 40 min of HIUS, the AFM data showed gradual decrease in median of cellulose fibers diameter with time of ultrasound treatment. It was, in particular, the result of thinning or segmenting the thickest fibers (Figure 2). This was indicated by a decrease in value of upper quartile (Q3) of the diameter distribution, which after 180 min of HIUS showed that the diameter of 75% of fibers was about half (5.7 nm) of the diameter of not-sonicated fibers (11.1 nm for 0.2% UT). Contrary to this, the values for Q1 and the minimal thickness of fibers (data not shown—1.3 nm on average) remained fairly constant, regardless of applied treatment. Much lower efficiency of ultrasound treatment in the case of relatively thin fibers suggested the presence of a lower size limit for cellulose, below which the applied ultrasound power did not cause further fragmentation of the fiber structure. Usually, the diameter of cellulose after HIUS treatment ranged from 3.6 nm for spinifex grass to even 100 nm for flax fiber [3]. However, in our previous research we showed that similar HIUS treatment to that used in this study caused disintegration of cellulose microfibrils to individual fibers with diameter of 2.68 nm for apple and 3.31 nm for carrot celluloses [7].

**Figure 3:**
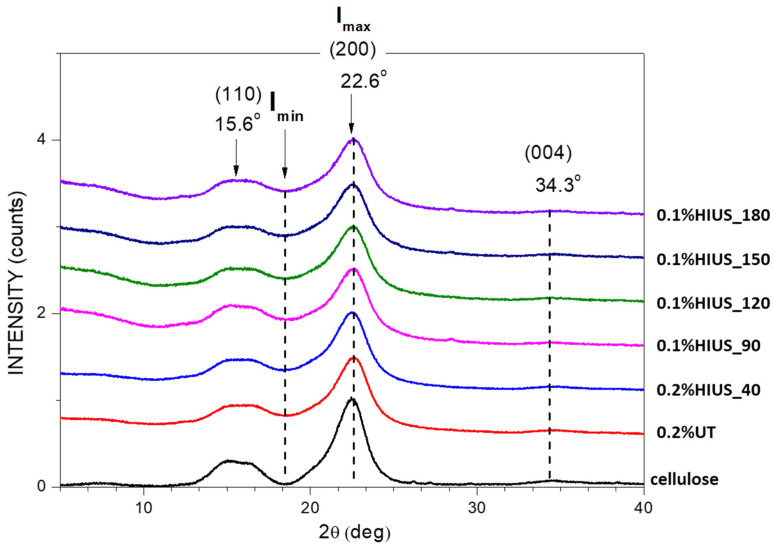
X-ray diffraction pattern of cellulose isolated for hop stems before (cellulose, 0.2%UT) and after HIUS treatment. The main diffraction reflections with Miller indices of dominant planes are denoted in the graph.

The X-ray diffraction patterns of investigated samples are presented in Figure 3. The most intensive reflections are those at 2Θ = 15° (110) and 22° (200), which are typical for cellulose I diffraction pattern. Moreover, the application of HIUS treatment did not cause changes in polymorph structure of cellulose. Previously, it was shown that the thermochemical method of cellulose isolation followed by HIUS treatment to obtain nanocellulose led to formation cellulose type II [7].

On the basis of X-ray diffraction pattern, the crystallinity degree and crystallite sizes of investigated samples were calculated. Isolated cellulose crystallinity degree was 67%, which is the average value for residues of crop plants such as wheat or rice straw, banana leaf and soy hulls [6]. Previously, Reddy and Yang (2009) showed that cellulose isolated from hop stem fibers had a crystallinity degree ca. 44%, which is significantly lower value than that obtained here [1]. 

The length of HIUS treatment time causes a slight decrease in crystallinity degree from 67% to around 60% after 2 h of HIUS (Table 2). The dropdown of crystallinity after the HIUS treatment is probably caused by damage of crystallites [18]. Additionally, the thickness of crystallites was calculated and for cellulose was 3.71 nm. The HIUS treatment for 2 h caused a decrease in crystallite thickness to 2.91–2.96 nm. These values are comparable with those obtained for cellulose and nanocellulose obtained from kraft pulp or fruit residues [7,9,19].

**Figure 4:**
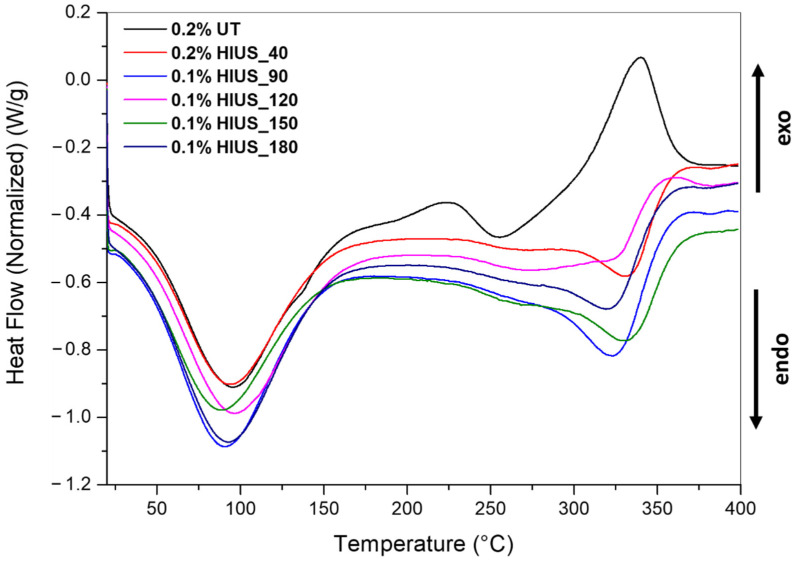
DSC curves of the studied cellulose after HIUS treatment.

The DSC curves are presented in Figure 4. Thermograms obtained for all samples presented an endothermic peak with a maximum between 84 and 97 °C connected with water loss from samples (Figure 4). Above the temperature of 150 °C, the two thermic processes for cellulose can occur: endothermic semimelting and exothermic decomposition. The second endotherm transition could correspond to the crystalline part of cellulose. Yeng et al., (2015) suggested that peak around 320 °C is probably connected with the breakage of glycosidic linkage in cellulose and partial decomposition of cellulose [20]. On the other hand, it was also shown that glycosidic linkage and decrease in polymerization degree can also occur in temperature ranges of 200–250 °C. The lower temperature of semimelting was related to more breakage of inter- and intramolecular bonds in cellulose, resulting in a decrease in crystallinity and endotherm transition [20]. Here, the lowest semimelting temperature was obtained for the least-processed sample 0.2%UT (Table 3). The decomposition peak was represented by exothermal transition, which occur at 339 °C and was shifted to higher temperatures for cellulose after HIUS treatment. The higher decomposition temperature means the higher thermal stability of cellulose.

**Figure 5:**
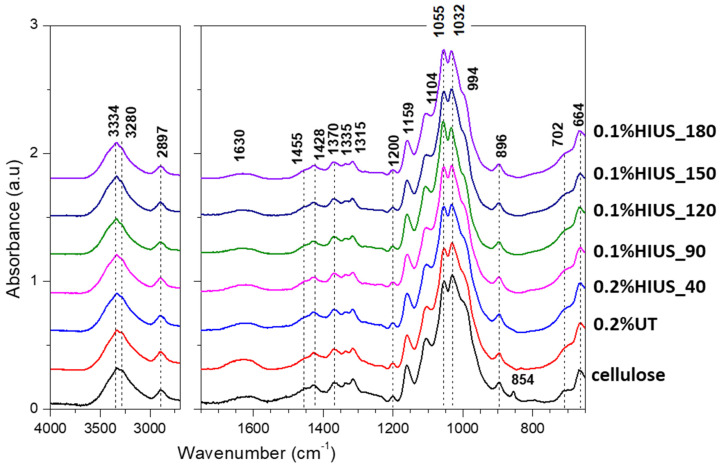
FTIR spectra of cellulose isolated from hop stems before and after HIUS treatment are presented in the range of 4000–2900 cm^−1^ and 1750–650 cm^−1^. The most characteristic bands are highlighted on spectra with dashed line.

FTIR spectra of cellulose are presented in Figure 5. The spectrum of isolated cellulose exhibits typical FTIR bands of cellulose 1428 and 1370 cm^−1^ (assigned to CH2 bending in cellulose and hemicellulose), 1159 and 1200 cm^−1^ (asymmetric and symmetric C–O–C stretching vibrations of glycosidic linkages, respectively), 1104 cm^−1^ (C-O and C-C stretching), 1055 cm^−1^ and 1032 cm^−1^ (C-O stretching), or 896 cm^−1^ (β-glycosidic linkage) [21,22]. However, spectrum of isolated cellulose also shows traces of lignins: 1455 cm^−1^ (aromatic skeletal vibration with C-H plane deformation) and 854 cm^−1^ (vibration of C-H in guaiacyl unit). Those bands diminish after application of HIUS treatment, which may be associated with lignin destruction [23]. Moreover, the obtained cellulose and nanocellulose were in an unoxidized state resulting in a lack of bands in the range 1750–1600 cm^−1^ (apart from a band at 1630 cm^−1^, characteristic for water). Since previous studies demonstrated that bleaching and ultrasound treatment could lead to oxidation of cellulose surface, one may conclude that hop stem cellulose is more resistant to oxidation by bleaching than apple and carrot parenchyma tissue studied before [7].

The band at 1428 cm^−1^ is also connected with vibration of intramolecular hydrogen bonds. No changes in this band means that HIUS did not change the intramolecular bands environment [24]. Another region responsible for hydrogen bonds in cellulose is the broad band from 3600 to 3000 cm^−1^. In particular, peaks in range 3500–3400 cm^−1^ reflect vibration of O2H⋅⋅⋅⋅O6 intramolecular bonding; peaks in range 3400–3310 cm^−1^ vibration of O3H⋅⋅⋅⋅O5 intramolecular bonding; while peaks in range 3310–3230 cm^−1^ reflect vibration of O6H⋅⋅⋅⋅O3 intermolecular bonding [19]. For cellulose isolated from hop stems, two peaks at 3334 cm^−1^ (O3H⋅⋅⋅⋅O5 intramolecular bonding) and at 3280 cm^−1^ (O6H⋅⋅⋅⋅O3 intermolecular bonding) could be recognized. The first one did not change under HIUS treatment, while the second clearly decreased. This result shows that during HIUS treatment the intermolecular bonds in cellulose are disrupted.

**Figure 6:**
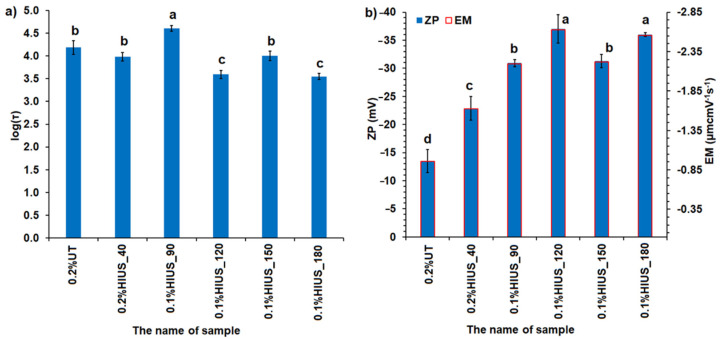
(**a**) The logarithm of the relaxation time marked as log(τ) as well as (**b**) zeta potential (ZP) and electrophoretic mobility (EM) of cellulose; bars indicate standard deviation; different letters represent statistically significantly different results (one-way ANOVA and the Tukey’ HSD post hoc test, *p* < 0.05).

Stability of cellulose dispersions was evaluated by relaxation time, zeta potential and electrophoretic mobility (Figure 6). Relaxation time τ corresponds to the size of dispersed particles, i.e., higher value of τ corresponds to bigger particles. Moreover, the interactions occurring between dispersed particles, such as cross-linking and gelation, extend the τ due to hampering the particles’ movement [25]. Process of cellulose sedimentation could be affected not only by the size of dispersed particles but also by their shape (defined often by aspect ratio), the fibers’ flexibility and entanglement as well as the ability for gel formation [26,27].

**Figure 7:**
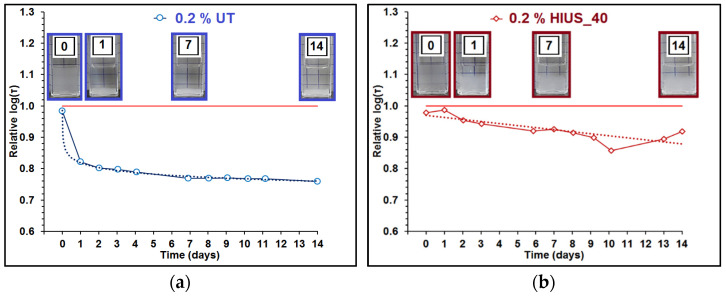

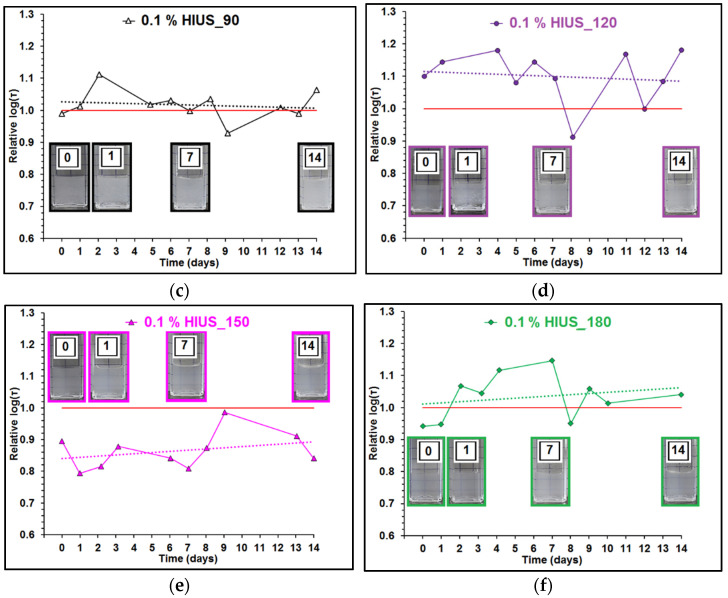
Relative log(τ) obtained during 14 days of experiment for (**a**) cellulose microfibrils (0.2% UT), (**b**) 0.2% dispersion of cellulose prepared using high-intensity ultrasonication for 40 min. (0.2% HIUS_40), and 0.1% dispersion of cellulose prepared using high-intensity ultrasonication for (**c**) 90 min. (0.1% HIUS_90), (**d**) 120 min. (0.1% HIUS_120), (**e**) 150 min. (0.1% HIUS_150), and (**f**) 180 min. (0.1% HIUS_180), respectively; red solid line marks relative log(τ) equal to 1; dotted lines show the fitted trend lines. The photographs of cuvette with cellulose dispersion are related to the consecutive days of measurements (0—initial day, 1—first day, 7—seventh day, 14—fourteenth day).

The longest relaxation time τ was obtained for sample 0.1%HIUS_90; intermediate ones for the samples 0.2%UT, 0.2%HIUS_40 and 0.1%, HIUS_150; and the shortest ones for the samples 0.1%HIUS_120 and 0.1%HIUS_180. The samples 0.1% HIUS_90, 0.1%HIUS_120, 0.1%, HIUS_150 and 0.1%HIUS_180 were characterized by the absolute values of ZP higher than 30 mV, which suggested their high stability. It was partially confirmed by the analysis of the relative log(τ) change with time (Supplementary Materials Figure S3 and Figure 7).

In the case of samples 0.1%HIUS_90 and 0.1%HIUS_180, both during the first 24 h (short storage, Supplementary Materials Figure S3) and after 14 days (long storage, Figure 7), the τ of dispersed cellulose was not significantly affected by the storage time. The value of relative log(τ) oscillated around 1. For sample 0.1%HIUS_120 the τ increased during the first hour (Supplementary Materials Figure S3) in respect to this recorded just after the sample preparation. However, during two weeks of investigations the value of relative log(τ) was independent of time (oscillated around 1.1). In the case of sample HIUS_150, τ decreased during the first hour, which could be connected with the occurring disaggregation. For the consecutive days, the relative log(τ) was on the level of 0.85.

Sedimentation of cellulose was clearly visible for samples 0.2%UT and 0.2%HIUS_40 (Figure 7). In the case of sample 0.2%UT, the value of relative log(τ) decreased significantly with the time of storage, which was described well by the power function (Supplementary Materials Figure S3). The gradient of cellulose concentration with the height of the liquid column in the cuvette was observed for sample 0.2%HIUS_40 after 24 h from preparation (Figure 7). The changes of relative log(τ) at this time were ambiguous (Supplementary Materials Figure S3c), but their analysis over the period of 14 days (Figure 7a) showed that τ decreased with an increasing storage time.

The obtained results clearly show that hop stems can be a valuable cellulose and nanocellulose source. The availability and amount of hop stems, which are rather considered as waste, cannot be also neglected. The isolated hop cellulose can be directly used in many new materials such as bioplastic composites. Most recently, the cellulose obtained from other agricultural wastes was used as reinforcing agent for PLA composites [28,29]. Other implementation of hop cellulose can be for composite panels used as insulators, as it was presented for phenol formaldehyde reinforced with rice straw cellulose or coir cellulose [30,31].

## 3. Materials and Methods

### 3.1. Materials 

Hop (*Humulus lupulus* cv Magnum) stems were obtained from Agricultural Experimental Plant “Jastków” Sp. z o.o. (Jastków, Poland) in September of 2019. Stems were freshly harvested and initially cut into 5–10 cm long pieces with pruning scissors and then fragmented in a mechanical cutting mill. All chemicals were purchased from Sigma Aldrich with purity of analytical grade.

### 3.2. Characterization of Hop Stems

The dry matter content was determined. Shredded and mixed samples (approximately 3 g) were dried (SUP-30W, Wamed, Warsaw, Poland) at 105 °C to constant mass. Dry matter content (DM) was calculated as DM = (m_2_/m_1_) × 100, where m_1_ was the mass of the fresh sample and m_2_—mass of the dried sample.

Van Soest analysis, with some modifications as it was described in detail previously, was used for cellulose, hemicelluloses and lignin determination [9,12,14,32]. This method enables separation of plant fractions by using two detergents: a neutral detergent—ND solution (sodium dodecyl sulfate, EDTA, pH 7.0)—and an acidic detergent—AD solution (cetyltrimethylammonium bromide in 1 N H_2_SO_4_). The neutral detergent removes pectic polysaccharides, phenolic compounds, proteins and sugars (giving NDF fraction, m_NDF_). Then, in the second step of extraction, acid detergent removes hemicelluloses (giving ADF fraction, m_ADF_). Subsequently, the cellulose is solubilized by 72% sulfuric acid (giving ADL fraction, m_ADL_). Therefore, the hemicellulose (H), cellulose (C) and lignin (L) content can be calculated:

H[g/100 g] = (m_NDF_ − m_ADF_)/m_SAMPLE_ × 100, (1)

C[g/100 g] = (m_ADF_ − m_ADL_)/m_SAMPLE_ × 100, (2)

L[g/100 g] = m_ADL_/m_SAMPLE_ × 100, (3)

where the cellulose (C%), hemicellulose (H%) and lignin (L%) content is expressed as a g/100 g of dry sample fraction (m_sample_—weight of dry sample). The content of neutral detergent soluble (NDS) composed of interalia of pectic polysaccharides, phenolic compounds and proteins can be also determined from the following formula:

NDS [g/100 g] = (m_SAMPLE_ − m_NDF_)/m_SAMPLE_ × 100 (4)

Additionally, the Fourier transform infrared spectrum (FTIR) of hop stems was obtained. Hence, hop stems were dried at 40 °C and ground using a ball mill (Retsch MM400) at 20 Hz for 20 min. The procedure of FTIR measurement was the same as for cellulose and nanocellulose, which is described below.

### 3.3. Isolation of Cellulose and Preparation of Nanocellulose

Cellulose was isolated by a modified version of the thermochemical method described by Szymańska-Chargot et al. [9]. Approximately 150 g of shredded hop stems was boiled in distilled water (3 L) for 15 min and then filtrated. In this step, cytoplasm, monosaccharides, starch and low molecular weight polysaccharides are removed. Then, 3 L of 1 M hydrochloric acid solution (HCl) was added to the residue and stirred by a magnetic stirrer for 30 min at 85 °C, and after that time the residue was filtered. This step was repeated with 0.5 M HCl in order to remove some acid polysaccharides. Afterward, the residue was stirred in 3 L of 1 M sodium hydroxide solution (NaOH) for 30 min at 85 °C; subsequently, the residue was filtered. This step was repeated threefold. The alkali treatment leads to removal of hemicelluloses and also to loosen bonds between cell wall polymers. The next step involved the bleaching of residue with 1–2% sodium hypochlorite solution for 60 min at 95–96 °C. This stage was repeated threefold. The resulting precipitate was cellulose, which was washed several times with hot deionized water until a neutral pH of the filtrate was obtained. 

The nanocellulose was isolated from hop stem cellulose via an ultrasonication treatment with a Sonics Vibracell ultrasonic homogenizer with maximum power output of 130 W (VCX-130FSJ; Sonics & Materials, Inc., Newtown, CT, USA). A 250 g portion of a 0.2% water suspension of hop stems cellulose was prepared. First, an Ultra-Turrax (T18 basic Ultra-Turrax, IKA, IKA-Werke GmbH & Co. KG, Staufen im Breisgau, Germany) instrument was used for 60 min high-speed blending to disrupt fibrils and initially disperse the obtained suspensions to obtain cellulose microfibrils (0.2%UT cellulose). The dispersed samples were then subjected to high-intensity ultrasonication for 40 min (0.2%HIUS_40). Afterward, the 0.2%HIUS sample was diluted to obtain a 0.1% weight (wt) dispersion. Each portion of 250 g of 0.1% was subsequently subjected to further high-intensity ultrasonication for 90, 120, 150 and 180 min, obtaining samples: 0.1%HIUS_90, 0.1%HIUS_120, 0.1%HIUS_150 and 0.1%HIUS_180, respectively. The sonication system contained a temperature probe, and to avoid heating the samples, an ice bath was used. The ultrasonic tip diameter was 6 mm with nominal amplitude of 120 μm. The operation amplitude of the ultrasonic homogenizer was maintained at 90% of the nominal amplitude. After preparation, all samples were refrigerated in 5 °C. 

### 3.4. Atomic Force Microscopy (AFM)

Sixty microliters of the aqueous suspensions of cellulose and nanocellulose samples were drop-deposited onto a 10 × 10 mm freshly cleaved mica base (EMS, Hatfield, PA, USA) and uniformly distributed using a Polos Spin150i spin coater (SPS-Europe B.V., Putten, The Netherlands). A Multimode 8 device equipped with a Nanoscope V controller (Bruker, Billerica, MA, USA) in automatic ScanAsyst mode (Bruker, Billerica, MA, USA) was used for imaging. A silicon tip on a nitride cantilever ScanAsyst AIR (Bruker, Billerica, MA, USA) with a nominal pyramidal tip radius of 2 nm and a nominal spring constant of 0.4 N m^−1^ was used. The experiment was performed in ambient air at a temperature of 20–22 °C and a relative humidity (RH) of 26–30%. The scanning area was 4 μm^2^ (aspect ratio of 1:1, 2 μm × 2 μm), while the scanning resolution was equal to 512 × 512 points. To maintain an appropriate quality of microscopic images, the scanning linear velocity was 0.9 Hz. For each sample, at least 9 images were collected. The heights of the AFM topographic images were analyzed using Matlab R2010a (MathWorks, Natick, MA, USA) by method previously described [7].

### 3.5. X-ray Diffraction 

Degree of crystallinity was determined by means of the X-ray diffraction (XRD) method. All samples were freeze dried before measurements. The X-ray diffractometer Empyrean (PANalytical, The Netherlands) was used. Samples were scanned with Cu Kα radiation (λ = 0.15418 nm). The parameters of the working lamp were as follows: U = 40 kV, I = 25 mA. The intensity of reflections was measured over the angular 5°–90° 2θ with step intervals of 0.05°. The duration of the reflection count was 10 s. On the basis of the recorded measurements, a mathematical model describing the relationship between intensity and 2θ was developed. The degree of crystallinity (CI_S_) was subsequently calculated according to the Segal method:

CI_s_ = (I_002_ − I_am_)/I_002_ (5)

where I_200_ is the intensity value for the crystalline cellulose (2θ = 22.5°), and I_am_ is the intensity value of the amorphous cellulose (2θ = 18°) [33,34]. The average thickness of the cellulose crystallites was estimated from the X-ray diffraction patterns using Scherrer’s equation:

d_hkl_ = Kλ/FWHM cosθ, (6)

where dhkl is the crystallite dimension in the direction normal to the hkl lattice planes; K is the correction factor, which is usually 0.9; λ is the radiation wavelength; θ is the Bragg angle corresponding to the (200) plane at 22°; and FWHM is the peak width at half the maximum intensity measured at the 22° peak.

### 3.6. Thermal Properties—Differential Scanning Calorimetry (DSC)

DSC analysis was performed with a TA Instruments DSC 250 (Waters, DE, USA) instrument using 10 mg of lyophilized sample (stored in a desiccator before measurement) sealed in aluminum pans and a 20–400 °C heating increase under a nitrogen flux of 50 mL min^−1^; the heating rate was 10 °C min^−1^. The data were analyzed using Trios v.4.2.1 (TA Instruments, Waters, DE, USA) software.

### 3.7. Fourier Transform Infrared Spectroscopy

Fourier transform infrared spectroscopy (FTIR) spectra were collected via a Nicolet 6700 FTIR spectrometer (Thermo Scientific, Waltham, MA, USA), and the Smart iTR attenuated total reflection (ATR) sampling accessory was used. Freeze dried samples were placed directly on ATR crystal and measured. The spectra were collected over the range of 4000–650 cm^−1^. For each material, 3 samples under the same conditions were examined. For each sample, 200 scans at a spectral resolution of 4 cm^−1^ were averaged. For a given material, the final average spectrum was then calculated. These spectra were normalized to 1.0 at 1030 cm^−1^ (bending vibration of ß-glycosidic linkage vibration in cellulose). All spectral manipulation was carried out using Origin Pro 8.5 (version 8.5 PRO, OriginLab Corporation, Northampton, MA, USA).

### 3.8. Zeta Potential and Sedimentation of Cellulose and Nanocellulose Dispersions

Sedimentation of cellulose particles dispersed in water (0.1%) was investigated using Zetasizer Nano ZS apparatus (Malvern Ltd., Malvern, UK). The same volume (1.3 mL) of each initially mixed suspension was placed in the cuvette. At the beginning, the measurements of relaxation time τ (back dynamic light scattering method (ISO 22412, 2017)) and electrophoretic mobility EM (laser Doppler electrophoresis method) [35] were performed in four repetitions. The Henry’s equation with the Smoluchowski approximation was applied for calculation of the zeta potential (ZP) of cellulose dispersed in water [36]. Next, the cuvette was tightly closed with a plug, and parafilm and was placed in the apparatus cell to measure τ at the various time intervals throughout the one day (temperature of 20 °C). Further measurements of τ were taken over the next 13 days. The samples were stored at a constant temperature (21 ± 1 °C). The logarithm of τ (µs), expressed as log(τ), and a relative log(τ), which was the ratio of log(τ) at a given time to log(τ) obtained for the freshly prepared sample, were studied. The obtained results were analyzed using Statistica 13.1 software (StatSoft Poland Ltd., Cracow, Poland).

## 4. Conclusions

Cellulose from hop stems was isolated by thermomechanical method and then underwent further disintegration by high-intensity ultrasonication which led to obtain nanocellulose in a form of stable dispersion. The proposed isolation method allows one to obtain about 29% of cellulose content in the dry mass of hop stems. This content is slightly lower compared with other agricultural byproducts; nevertheless, different methods of isolation should be considered in this comparison. However, as proposed in this study, isolation protocol allows isolation of relatively highly crystalline cellulose (67%) that is similar to other agricultural byproducts. High-intensity ultrasonication performed on isolated cellulose allows for tuning crystallinity degree, diameter of fibers and stability of the nanocellulose dispersions. The most intensive ultrasonication (180 min) allows one to obtain very stable dispersions. Our results also suggest that high-intensity ultrasonication changes intermolecular hydrogen bonds, while intramolecular bonds are not affected, which is the reason for changes in nanocellulose size distribution. Furthermore, the thermal stability of cellulose after HIUS treatment is higher. In summary, we postulate that hop stems as unutilized byproducts may be partially valorized to cellulose and nanocellulose that may be further used as a natural reinforced or packaging biocomponent. 

## Figures and Tables

**Table 1:** The content of neutral detergent soluble (NDS), neutral detergent fiber (NDF), hemicellulose (H), cellulose (C) and lignin (L) in hop stems.

NDS	NDF	H	C	L
g/100 g d.m.	g/100 g d.m.	g/100 g d.m.	g/100 g d.m.	g/100 g d.m.
**51.07 ± 0.51**	**48.93 ± 0.51**	**10.94 ± 0.46**	**29.06 ± 0.86**	**8.93 ± 0.40**

NDS—neutral detergent soluble, NDF—neutral detergent fiber, H—hemicellulose, C—cellulose, L—lignin, d.m.—dry matter.

**Table 2:** The crystallinity index and thickness of crystallites of cellulose isolated from hop stems after different duration of HIUS treatment.

	X_c_%	d_hkl_
	(%)	(nm)
**cellulose**	67.2	3.71
**0.2%UT**	68.4	3.20
**0.2%HIUS_40**	66.9	3.10
**0.1%HIUS_90**	58.7	3.01
**0.1%HIUS_120**	60.4	2.96
**0.1%HIUS_150**	61.3	2.91
**0.1%HIUS_180**	60.4	2.94

**Table 3:** The crystallinity index and thickness of crystallites of cellulose isolated from hop stems after different duration of HIUS treatment.

	SEMIMELTING		DECOMPOSITION	
	T_max_	Enthalpy	T_max_	Enthalpy
	(°C)	(J/g)	(°C)	(J/g)
**0.2%UT**	255.60	20.354	339.86	85.841
**0.2%HIUS_40**	333.58	25.457	361.73	13.601
**0.1%HIUS_90**	323.88	37.785	358.94	14.028
**0.1%HIUS_120**	323.88	22.700	353.56	21.663
**0.1%HIUS_150**	331.83	21.747	364.56	11.422
**0.1%HIUS_180**	321.77	25.870	358.78	25.870

## Data Availability

Not applicable.

## Supplementary Materials

The following supporting information can be downloaded at: https://www.mdpi.com/article/10.3390/molecules27092624/s1: Figure S1. Photos of cellulose isolated from hop stems (a) and dispersions of cellulose after mechanical disintegration (0.2%UT) and 180 min of HIUS treatment (0.1%HIUS_180) (b). Supplementary Materials Figure S2. Representative atomic force microscope (AFM) height images of cellulose isolated from hop stems after different duration of HIUS treatment. The image dimension is 2 µm × 2 µm. Supplementary Materials Figure S3. The values of relative log(τ) obtained for particular sample (a–f) during the first 24 h of experiment; solid line marks relative log(τ) equal to 1; dotted line shows the fitted trend line.

## Sample Availability

Not available.
