# Supplementary material for: The Influence of High-Intensity Ultrasonication on Properties of Cellulose Produced from the Hop Stems, the Byproduct of the Hop Cones Production
Source: Molecules. 2022 Apr 19;27(9):2624. doi: 10.3390/molecules27092624 (PMC9102265; doi:10.3390/molecules27092624)
Supplement: Supplementary file 1 [file molecules-27-02624-s001.zip › molecules-1672730-supplementary.pdf]

molecules

MDPI

Supplementary material

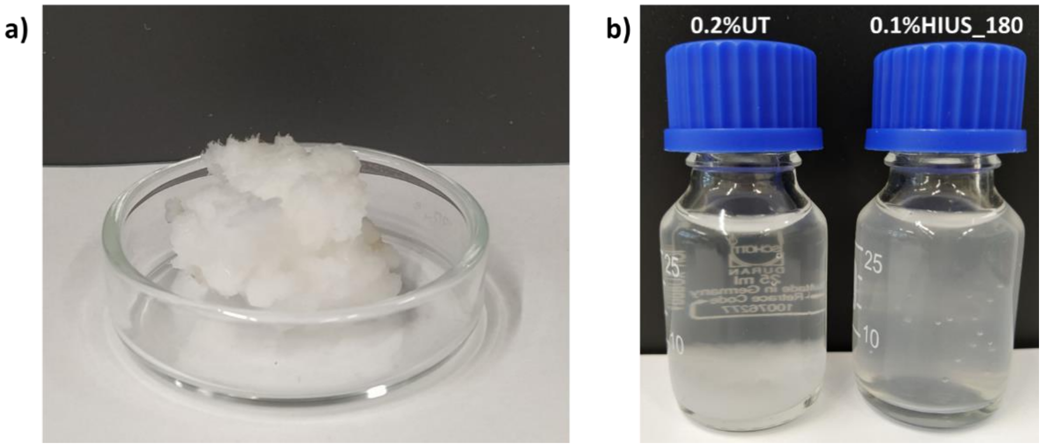

Figure S1. Photos of cellulose isolated from hop stems (a) and dispersions of cellulose after mechanical disintegration (0.2%UT) and 180 min of HIUS treatment (0.1%HIUS_180) (b).

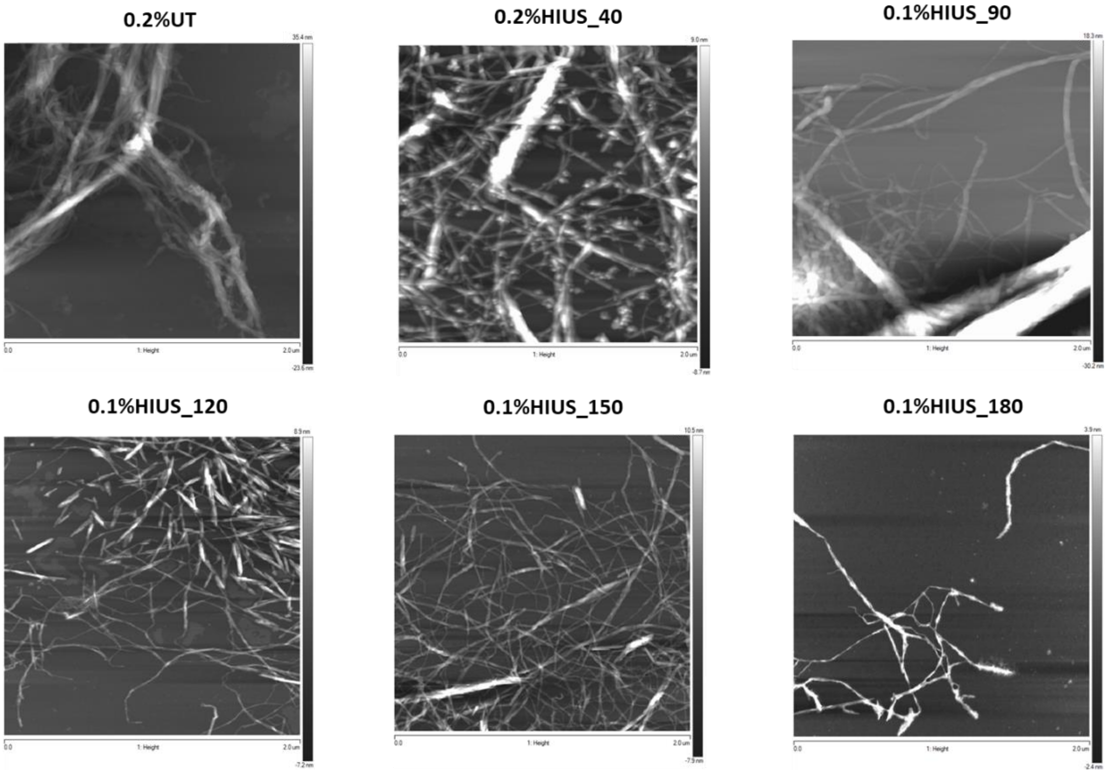

Figure S2. Representative atomic force microscope (AFM) height images of cellulose isolated from hop stems after different duration of HIUS treatment. The image dimension is 2μm x 2μm.

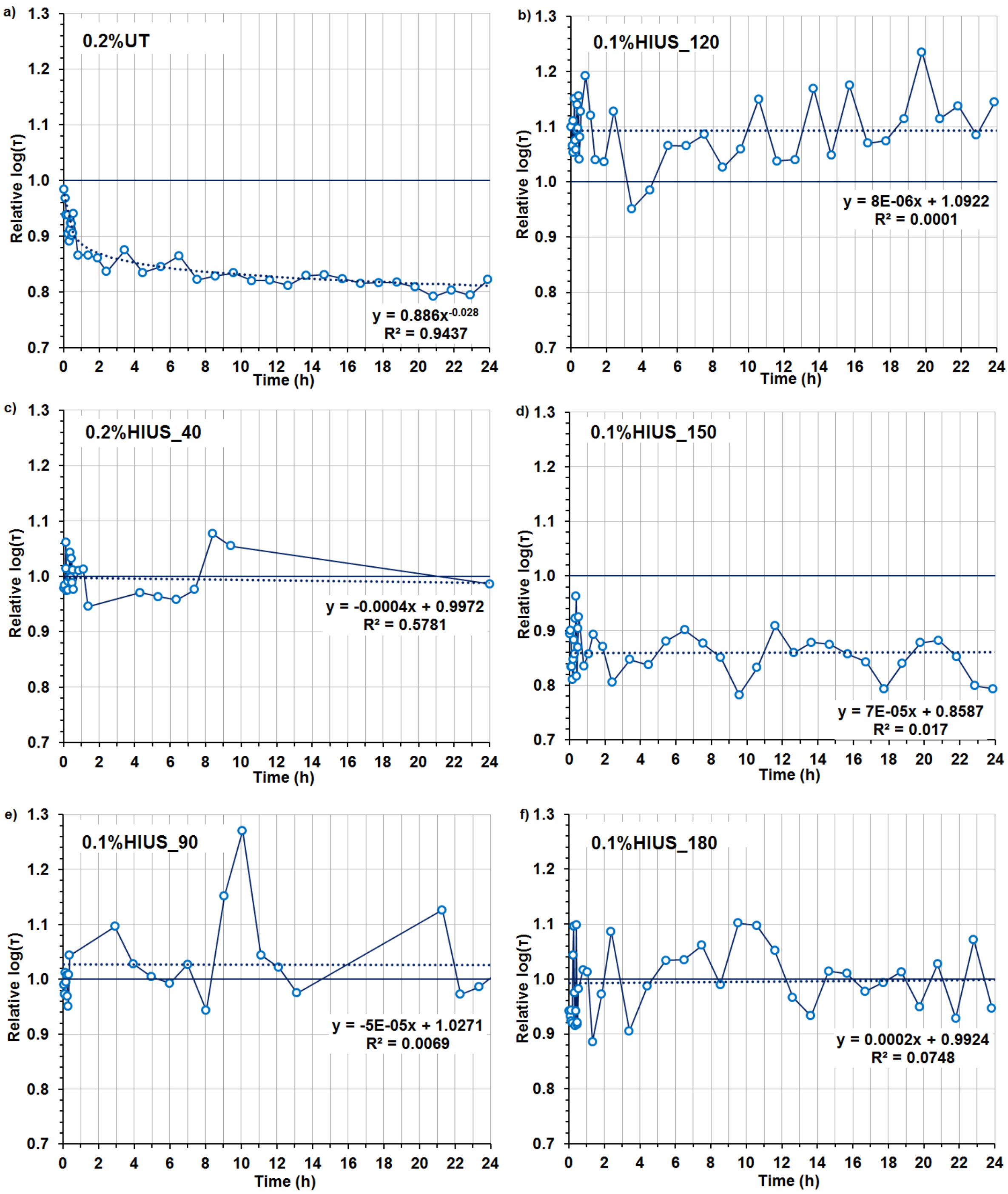

Figure S3. The values of relative log(τ) obtained for particular sample **(a-f)** during the first 24 hours of experiment; solid line marks relative log(τ) equal to 1; dotted line shows the fitted trend line.
